# Barriers to access, pathways to equity: clinicians’ perspectives on mental health service delivery

**DOI:** 10.1186/s12913-025-13948-3

**Published:** 2026-01-08

**Authors:** Emma Peddigrew, Kaya Costanzo, Sienna Armstrong, Celine Huang, Tasmia Hai

**Affiliations:** 1 Department of Occupational Science and Occupational Therapy, University of Toronro, Toronto, ON Canada; 2 Department of Educational and Counselling Psychology, McGill University, Montreal, QC Canada; 3 Department of Psychology, McGill University, Montreal, QC Canada; 4 Interfaculty Program Cognitive Science, Faculty of Arts and Sciences, McGill University, Montreal, QC Canada

**Keywords:** Clinicians, Socioculturally diverse, Mental health, Cultural responsiveness

## Abstract

**Background:**

This study explored clinicians’ perceptions of the barriers that immigrant, refugee, and ethnically diverse populations face in accessing mental health (MH) care in Canada, the challenges clinicians encounter in service delivery, and their recommendations for improving access and quality of care.

**Methods:**

Guided by a practice-based research approach, a qualitative descriptive approach was used, employing semi-structured interviews and thematic analysis. The study was conducted across community-based MH organizations in multiple Canadian provinces within primary and community care settings. Nineteen MH clinicians, including social workers, psychotherapists, and counselors were purposively sampled. Eligible participants were currently employed in MH roles and had direct experience working with diverse populations; there were no formal exclusion criteria.

**Results:**

Clinicians identified two overarching categories of barriers: logistical challenges (such as long wait times, cost, and limited access to multilingual services) and cultural/social barriers (including stigma, cultural mismatch, and mistrust of Western MH models). Clinicians also reported institutional challenges, such as limited training and resources for culturally responsive care. Recommendations focused on enhancing cultural responsiveness, increasing clinician education, and implementing system-level changes to reduce structural inequities.

**Conclusions:**

Clinician insights highlight the need for more accessible, equitable, and culturally responsive MH services for diverse populations in Canada. These findings have implications for future research, clinician training, and policy reform.

**Supplementary Information:**

The online version contains supplementary material available at 10.1186/s12913-025-13948-3.

## Barriers to access, pathways to equity

Canada’s population is increasingly diverse, with nearly one-quarter identifying as immigrants [1]. Ethnic minorities, immigrants, and refugees often face heightened stress and trauma due to displacement, discrimination, unemployment, and lower socioeconomic status [2, 3]. Addressing their mental health (MH) needs is complex, as many psychotherapeutic programs rooted in Western frameworks fail to provide effective support [4]. It is important to note that “socioculturally diverse” encompasses a broad spectrum of clients—including those from both Western and non-Western contexts—whose intersecting identities shape how they experience and access care [5]. Socioculturally diverse populations are often distinguished from dominant or historically privileged groups based on factors such as ethnicity, race, language, religion, socioeconomic status, gender, and education [5]. The term, “socioculturally diverse” recognizes the intersectionality of these identities and extends “newcomer” or “immigrant” to include a broader range of social determinants beyond nationality or citizenship. Numerous studies have identified barriers to MH care among socioculturally diverse populations, including a lack of cultural humility among providers, stigma, and structural obstacles like economic and linguistic barriers [3, 6–9]. While the barriers to MH care for socioculturally diverse groups are known, research on solutions remains limited, particularly from the perspective of clinicians who are actively practicing and navigating these barriers regularly.

### Barriers to MH service utilization and access

#### Lack of cultural humility and mistrust

Cultural humility is defined as “having an interpersonal stance that is other-oriented rather than self-focused, characterized by respect and lack of superiority toward an individual’s cultural background and experience” [10]. Conversely, a lack of cultural humility—manifesting as rigid adherence to Western-centric models, unexamined biases, or limited understanding of clients’ cultural contexts—has been shown to undermine therapeutic relationships and reduce service utilization among diverse populations [8–11]. Culturally humble clinicians are empathetic, open to learning about clients’ identities, and conscious of their own biases and privilege, all of which enhance the therapeutic process [11]. Research indicates that clients’ perceptions of cultural humility in therapy are associated with stronger therapeutic alliances and improved therapy outcomes [9, 10]. Mistrust toward the MH care system is another significant barrier faced by socioculturally diverse groups [8, 12]. Mistrust, rooted in historical mistreatment and systemic inequities, often deters culturally diverse individuals from seeking MH care, contributing to service underutilization [7].

#### Stigmatization of MH

Stigma surrounding MH is rooted in cultural norms and values across both Western and non-Western societies [13]. Stigma can take multiple forms: internalized stigma, where individuals adopt negative beliefs about themselves; social stigma, reflected in others discriminatory attitudes and behaviors; structural stigma, embedded in policies and institutional practices; and stigma from MH providers themselves, which can affect engagement and quality of care [14]. Research suggests that societal and provider-level stigma can deter help-seeking, even when individuals hold positive personal attitudes toward MH support [6]. Internalized stigma, for instance, can elicit shame and exacerbate symptoms [15]. Recognizing these distinctions clarifies potential intervention targets, from community education to organizational and provider-level training, to reduce barriers to MH care.

#### Structural barriers

Structural barriers to MH care stem from how services are organized and delivered [16]. These include economic, linguistic, and logistical challenges, as well as a lack of culturally responsive programs. High therapy costs and limited funding for community-based MH organizations hinder access for culturally diverse populations [3, 8, 17]. Furthermore, language is a significant structural barrier to MH care accessibility [3]. Many culturally diverse individuals may lack proficiency or comfort in the therapist’s language, making services less accessible. Kirmayer and colleagues emphasize the role of interpreters and “cultural brokers” in bridging these gaps [16]. While interpreters facilitate communication, challenges include translating nuanced meanings and potential shifts in therapy dynamics. Cultural brokers offer a more integrative approach, incorporating both cultural perspectives to enhance understanding between clients and clinicians [18]. Additionally, Western-based psychological models may not resonate with non-Western clients, limiting effectiveness [4]. Without culturally responsive programs, MH providers may struggle to address the unique needs of diverse clients, potentially exacerbating MH issues [3]. Long waitlists and transportation issues further restrict access, especially in underserved areas [3, 8]. Despite some efforts toward adaptation, the lack of culturally responsive services remains a significant barrier.

### The value of clinicians’ perspectives- practice-based research

Many service providers struggle to integrate evidence-based research into daily practice [19], partly due to the controlled and artificial environments in which psychotherapeutic programs are evaluated in research settings [20]. In addition, front-line clinicians do not always have access to up-to-date evidence-based information due to time constraints, implementation issues and cost of having access to academic journals [21]. Specifically, some clinicians have reported that psychotherapeutic programs miss contextual information required for external validity [21]. Given the individualized nature of therapy, this can result in treatment programs that fail to meet clients’ needs, particularly for socioculturally diverse populations whose unique experiences are underrepresented in such research. To bridge this gap between research and real-world practice, this study employed a practice-based research (PBR) approach, which emphasizes the iterative integration of clinical expertise, patient needs, and contextual realities [22]. Practice-based research prioritizes clinicians’ firsthand experiences as the foundation for generating theory and shaping psychotherapeutic treatment programs. This orientation helped ensure that findings would be directly relevant to service delivery by embedding research within the context of clinical practice. By incorporating real-world clinical insights, practice-based research can help make MH services more inclusive, adaptable, and culturally responsive.

## The present study

The barriers to accessing MH care among socioculturally diverse groups are widely recognized. However, there is limited research capturing the subjective lived experiences of clinical professionals in treating such clients. Given the critical role clinicians play in shaping MH service delivery, their insights are essential for identifying systemic gaps and developing culturally responsive solutions. This study aims to explore clinicians’ perceptions of the barriers diverse groups face in accessing MH care, the challenges clinicians encounter in providing services, and their recommendations for improving service delivery and accessibility. The following questions guided the study:

1. What systemic and structural barriers do clinicians identify as limiting access to MH care for socioculturally diverse groups?
2. What challenges do clinicians face in providing MH services to these populations?
3. What recommendations do clinicians offer to improve accessibility and delivery for socioculturally diverse communities?

## Methods

A qualitative descriptive approach was utilized to capture the nuances of clinicians’ experiences while prioritizing participants’ voices and reflexivity to minimize researcher bias. Aligned with a practice-based research framework [22], the study foregrounded clinician expertise as a form of knowledge generation, emphasizing the practical relevance of findings for everyday service provision. Qualitative methods were chosen for their capacity to highlight lived experiences that might otherwise be overlooked in quantitative research [23]. This approach ensured that participants’ narratives remained central to the findings, allowing for an authentic representation of their insights [24]. The study received ethical clearance from the [BLINDED] Research Ethics Board, and all participants provided informed consent prior to data collection.

### Recruitment and participants

A total of 35 community-based mental health agencies across Canada that explicitly supported individuals from diverse socio-cultural backgrounds were identified through online searches and contacted for recruitment. The study was also advertised on a Listserv created by the Division of Transcultural Psychiatry at McGill University, which facilitates discussion on culturally informed mental health practices worldwide. Organizations distributed recruitment materials to all clinicians, and interested individuals completed an online eligibility screener. Eligible participants were Canada-based mental health clinicians who primarily supported socioculturally diverse clients. A snowball sampling strategy was used to identify additional eligible participants, particularly those less accessible through conventional recruitment methods [25]. At the beginning of each interview, a broad definition of “equity-deserving” was provided to ensure participants clearly understood the study’s purpose. Interviews were conducted between February and September 2024, continuing until data saturation was reached. The demographic form capped experience at “7 + years” for consistency among participants with extensive practice. Demographic details are presented in Table 1. All participants were women, reflecting a self-selected sample rather than the study design. Recruitment emails were sent to multiple agencies and distributed to all clinicians; however, only women volunteered to participate. This may limit the generalizability of findings to clinicians of other genders.

**Table 1:** Participant demographics

Code	Pseudonym	Age	Gender	Race/Ethnicity	Years of Experience	Professional Designation
01	AM	61+	Woman	White European	7+	Clinical Social Worker
02	NS	45–50	Woman	Middle Eastern	3–4	Somatic Practitioner
03	YJ	41–44	Woman	Black African	7+	Psychologist/Psychological Associate
04	KT	31–35	Woman	South Asian	3–4	Psychologist/Psychological Associate
05	LM	31–35	Woman	Middle Eastern	7+	Social Worker, Psychotherapist, Couple/Family Therapist
06	RJ	36–40	Woman	South Asian	7+	Physician (MD)
07	LM	41–44	Woman	East Asian	3–4	Psychotherapist
08	TH	45–50	Woman	Black African	7+	Psychologist/Psychological Associate
09	LD	45–50	Woman	Black Caribbean	7+	MH Clinician
10	JD	41–44	Woman	Middle Eastern	7+	Physician (MD)
11	SM	56–60	Woman	White European	7+	Art Therapist
12	GM	26–30	Woman	White Canadian	3–4	Social Worker
13	DR	31–35	Woman	Middle Eastern	< 1	Social Worker
14	LS	61+	Woman	South Asian	7+	Physician (MD)
15	JP	36–40	Woman	East Asian	5–6	Social Worker (MSW/BSW)
16	NT	45–50	Woman	White European	7+	Psychologist/Psychological Associate
17	AN	45–50	Woman	South Asian	7+	Psychotherapist
18	JX	51–55	Woman	East Asian	7+	Social Worker/ Psychotherapist
19	EL	45–50	Woman	East Asian	5–6	Social Worker

### Data collection

After confirming eligibility and obtaining consent, participants completed an online demographic survey before participating in semi-structured interviews via Microsoft Teams. Each 60-minute session was audio-recorded and transcribed. As part of a broader study on clinicians’ experiences, discussions covered professional background, service accessibility, systemic challenges, and areas for improvement. The current analysis specifically focused on clinicians’ perspectives on barriers to serving diverse populations and their recommendations for enhancing service delivery. Sample questions included:

- What logistical and economic barriers have you encountered while supporting clients from diverse backgrounds?
- How would you recommend continuing engagement with clinicians who support diverse community members in the future?

A comprehensive list of interview questions is available in the supplemental materials.

### Data analysis

Interview recordings were transcribed using Microsoft Teams, de-identified, and reviewed for accuracy by two research members. An inductive thematic analysis [23] was conducted using NVivo 14.0 (QSR International) to systematically code and identify patterns across participants’ narratives. Initial coding was conducted independently by two researchers, who then met to compare and consolidate codes through discussion. A third senior team member reviewed the preliminary codebook, which was refined iteratively over several rounds of collaborative analysis. Peer debriefing and documentation of analytic decisions supported transparency and credibility. Throughout the analysis, researchers engaged in reflexive memo-ing to document personal assumptions, potential biases, and interpretive decisions, ensuring that findings remained grounded in participants’ perspectives. Although traditional quality criteria (credibility, dependability, confirmability, transferability) were not explicitly named, rigor was maintained through independent coding, iterative codebook refinement, peer debriefing, and documentation of analytic decisions [26]. Data saturation was considered reached when no new themes emerged in later interviews [26].

## Results

Three major themes emerged from the participants’ interviews (see Table 2). The themes are presented below, supplemented by excerpts from clinician testimonies that provide valuable insights into these challenges. The results conclude with clinicians’ recommendations for training, offering important considerations for improved equity-driven care.

**Table 2:** Major themes of interviews

1 Logistical Barriers
1.1. Financial Constraints
1.2. Wait Times
1.3. Transportation
1.4. Language
2 Cultural and Social Barriers
2.1. MH Stigmas
2.2. Mistrust of MH Service
2.3. Family and Community Expectations
2.4. Confidentiality
3 Clinician Recommendations
3.1. Culturally Responsive Care and Bureaucracy
3.2. Shifting Approaches to MH Treatment
3.3. Self-Care and Ongoing Cultural Learning

## Logistical barriers

### Financial constraints

Most clinicians (14 of 19) identified financial constraints as a significant barrier to accessing MH services, particularly for marginalized or underserved populations. Many clients struggle to afford treatment, resulting in delayed or foregone care.

*Not everybody is able to pay. I reserve a certain percentage to clients who cannot pay…sometimes for free*,* sometimes for a lower fee. (NT)*

*We have to work on gaining more government resources*,* financially we need to find a way because most of the persons are vulnerable and not able to pay for services. (EL)*

Several clinicians explained that financial constraints also impact providers, with low salaries discouraging some from working with diverse populations:

*We don’t have enough money to hire [people]. My salary is moderate*,* but fortunately I’m not looking for money and I think it’s OK for me to have this kind of life. (SM)*

*It is a big point of contention at the larger agency that I work for*,* that psychologists are severely underpaid*,* even within the developmental sector in Toronto. (KT)*

Clinicians noted that limited funding in organizations serving diverse populations hinders support for at-risk clients.

*We’re only funded for a certain number of positions and have to think about caseload of those staff*,* we don’t want to overload them…funding is a barrier. (TC)*

*[We need] more funding to support some of these… diverse populations that we’re trying to support. (AM)*

*We had three leaders*,* we had constant burnout. Wonderful staff but terrible cases and very underfunded. (LS)*

Clinicians highlighted the interconnected nature of financial challenges, where limited funding affects both the availability of services for clients and the sustainability of their own professional work. These dual pressures emphasize the urgent need for systemic financial reforms to enhance accessibility and capacity.

### Wait times

Long wait times were consistently cited as a major issue, often delaying access to essential services due to high demand and insufficient staffing:

*I have to tell my clients to wait because it’s fully booked…we don’t have enough people. (LM)*

*It is equal for everyone on the wait list*,* it is 14 months. We’re trying to find solutions. (GM)*

Delays can result in clients disengaging from the system or seeking alternative, sometimes inappropriate, support:

*There are gaps where it’s not counselling they need*,* but daily support*,* but to request for that type of support*,* it’s a long wait*,* and sometimes by the time they get it*,* it’s too late. (LK)*

*Not everybody understands the service*,* so when they don’t get a call right away*,* they would move on… there’s a lot of falling through the cracks. (TH)*

*By the time clients get a call for services*,* their situation has changed and they’re no longer willing to look for help. (SM)*

Wait times are a consistent issue across organizations and can sometimes prevent clients from accessing services altogether. These insights reveal the resource shortages clinicians face and the need for change.

### Transportation

Clinicians noted the lack of affordable and accessible transportation frequently limits clients’ ability to receive services.

*We don’t have the capacity to go to the client*,* they have to come here*,* by bus*,* or a metro*,* but it’s not free*,* it’s not super easy to get to. (GM)*

*So often*,* for them it’s not being able to easily get downtown for example*,* perhaps because of lack of access to a vehicle or transit is impossible to navigate. (KT)*

*If you live in an area where you don’t have any provider and have to travel for an hour to speak to a clinician…that’s too far. (TC)*

Some clinicians make accommodations to help clients overcome transportation challenges, including offering virtual sessions or providing bus tickets.

*But for some people if they have kids and they can’t come*,* I will be on zoom*,* I will provide online sessions too if they ask. (GN)*

*We do provide bus tickets*,* but not on an ongoing basis. One would have to figure out a way to sustain their own sort of transportation. (LM)*

These reports illustrate ongoing barriers to accessing services due public and private transportation costs, accessibility issues related to public transportation, or limited time to travel to the clinician’s office.

### Language

Language barriers represent a critical impediment for clients from linguistically diverse backgrounds, especially for newcomers to Canada. Clinicians reported that limited English or French proficiency often complicates clients’ ability to navigate services and communicate their needs effectively:

*Linguistic barriers have come up*,* often because it also then can translate to challenges around awareness of services or how to navigate certain services. (KT)*

*The language barrier is a lot. It’s a very big problem for them*,* so they will be frustrated*,* they will be feeling the difference*,* the culture shock and the sadness and the grief of who they were back home. (GN)*

*It starts a lot with language in Quebec. Some people still have issues to find even services in English let alone other languages. (AK)*

Even when interpretation services are available, clinicians noted that clients may feel uncomfortable or struggle to build rapport when an interpreter is present:

*We do have the capacity to bring in an interpreter. The only thing with that is it’s hard to sit with an interpreter and a client for 12 sessions and have the client feel comfortable. (EL)*

Conversely, some clinicians shared how their organization has a good capacity to provide services in different languages, which is appreciated by clients:

*We are diverse here. We have many people that speak Arabic*,* Ukrainian*,* Farsi*,* Dari. We have almost all languages*,* so if someone needs some help they can stop in and we’ll help. (GN)*

[Clients] say, “Oh you speak Arabic, I will be able to express myself.” (JD).

These findings demonstrate the need for expanded language services to address the linguistic diversity of clients effectively.

## Cultural and social barriers

### MH stigma

Clinicians noted that stigma and cultural differences shaped engagement. As some observed, how minority families either stigmatize these services or do not see the need for them, contrasting with the enthusiasm seen in French and English Canadian families.

*People from minority backgrounds*,* either stigmatize it or don’t see the need versus my French and English Canadian families*,* […] they get excited about it. (RA)*

*We still have stigma about mental health because many people don’t understand that mental health is not mental illness.* (YJ)

The stigma surrounding MH is more prominent among first-generation immigrants and individuals from racialized communities:

*We are seeing a lot of Canadian-born individuals feel more comfortable about getting therapy. But other communities like people of color*,* a lot of first-generation immigrants*,* they still feel uncomfortable. (JP)*

Shame and fear of judgement also hinder treatment access:

*I’ve seen more teenagers be worried their parents will find out they’re seeking services…teenagers of different backgrounds told me similar things*,* how mental health issues were not perceived as something you discussed with a stranger. (SM)*

*It will make them feel ashamed that*,* ‘I have something wrong*,* I need to admit that I am deficient’ in order to seek help. (JX)*

MH stigma is compounded by cultural differences in dealing with emotional issues:

*I think a lot of the times this population will access services and feel judged and stigmatized. They feel that within their own families. (NP)*

*The population I work with*,* they’re coming from a shame-based culture where it’s very different from Western [culture]. They don’t want anybody to know what happened. (NS)*

The concept of MH varies significantly across cultures, where help-seeking may be seen as a sign of weakness or shame rather than self-care:

*I’ve heard a lot from the Chinese population that they’re high achieving. They sacrifice a lot to send their kids to private school. The kids feel a sense of betrayal when they need mental health support. (SM)*

*In the Chinese community*,* mental health is still stigmatized. People only seek help when things are really bad. (JP)*

*For some*,* it’s very difficult to speak loud about their desires and what they’re feeling. For them*,* psychological issues are a sign of incompetence. (SM)*

The pressure to conform to cultural expectations often results in clients minimizing their MH needs, making them reluctant to seeking care.

### Mistrust of MH services

Cultural mistrust was particularly common when service delivery did not align with cultural expectations or when language barriers existed.

*The psychotherapy or counseling in translation to our language is not easy… people will think counseling here is casual*,* but for them*,* it will make them feel ashamed. (JX)*

There’s fear that accessing services may impact their immigration status or employment. (TH)

As AC stated, “they worry about their families being taken away, deportation, or imprisoned.” Mistrust often stems from previous negative interactions with authorities and fears of consequences that may arise from seeking help:

*If someone already experienced racism with providers*,* maybe at the hospitals*,* they have biases and it’s difficult to refer them to see a provider even if it’s necessary.* (YJ)

LD discussed this mistrust as a call to action:

*We talk about the history behind Western medicine…there’s a lot of mistrust…we need to engage the community and get their input for issues and solutions*,* instead of telling them what they need.*

The widespread impact of healthcare-related trauma in diverse populations highlights the need for education and action to halt this cycle of trauma and mistrust.

### Family and community expectations

Family and community expectations play a crucial role in shaping how clients perceive and respond to MH challenges:

*Culturally*,* there’s distance to get services for fear of parents being worried about reputation… my Chinese clients would tell me “My parents view mental health as a weakness.” There is a reluctance*,* and that reluctance is a defense mechanism. (SM)*

Furthermore, cultural expectations around success, family reputation, and the perceived shame of MH issues often create barriers to accessing support:

*Sometimes parents are not aware of mental health issues*,* they see it as a weakness or something to hide. (SM)*

AN shared times where individuals were reluctant to seek support due to feeling embarrassed, “they say, ‘I don’t want to get mental health services…that’s embarrassing.” Community pressures and cultural expectations thus become key social barriers, making it difficult for individuals to prioritize MH needs:

*My own Chinese community*,* a lot of people could benefit*,* but because of the stigma they hesitate about stepping into a therapy room. So how do we make sure that’s helping the community as a whole? (SM)*

*The population I work with*,* they’re coming from a shame-based culture where it’s very different from Western*,* it’s guilt-based…they don’t want anybody to know. (NS)*

Participants noted that clients from diverse populations are often influenced by loved ones’ opinions, creating a barrier to seeking MH treatment due to stigma.

### Confidentiality

Participants SM, TH and LM identified that shame can also create concerns around treatment confidentiality among clients from diverse backgrounds. RJ and SM shared that this issue is very prevalent with younger clients:

*They’ll often ask me right on the outset*,* “are you going to tell my parents?” or “I want to make sure you don’t tell my parents.” (RJ)*.

*I had a woman from a Chinese background*,* her father was against her seeking mental health services. There’s shame*,* what if her family knows. (SM)*

*The confidentiality between the parent and children is something I need to take a lot of time to explain. (LM)*

Concerns around confidentiality also occur when patients are afraid that their information will be shared among people across the organization:

*One of the concerns was if they come to seek a service*,* maybe an employment counselor*,* and that person refers them to us*,* they’re worried that whatever they share is going to be shared with the referral person. (TH)*

Despite an increase of MH awareness, socioculturally diverse individuals still face challenges, particularly when stigma and past trauma act hinder help-seeking. Insights from these clinicians offer profound recommendations to improve MH service access, equity, and inclusion.

## Clinician recommendations

### Culturally responsive care and bureaucracy

Many participants raised concerns about limited culturally responsive training in their education. Most participants (17 of 19) highlighted that their formal training lacked sufficient attention to cultural differences. GM noted, “we didn’t talk about cultural considerations” while others criticized their Western-centric training as ill-suited to the clients they serve:

*I don’t feel like there was enough classes or discussion around working with cultural difference…A lot of it was about theories created in the West and you couldn’t apply them to most of the clients I work with today. (NH)*

*[I wish training] prepared us to be open to new ideas*,* or perspectives about other cultures. (GN)*

*The training needs to be more about how to work with diverse populations. So many psychologists don’t do the work because they don’t feel equipped. (KT)*

Several clinicians linked these gaps to bureaucratic structures that limit flexibility and innovation in training and service delivery. Institutional priorities, standardized procedures, and funding restrictions were described as barriers to implementing culturally responsive care. As JD emphasized, “the organization and health system needs to be included in the training.” NT added that practical, hands-on experience is crucial for preparing future clinicians:

*While it’s important to have a structured training*,* I really feel the experience part*,* it’s a bit more difficult*,* but it’s really important to make sure trainees have actual experience.*

In addition to clinical training, some participants emphasized the need to train interpreters and cultural mediators who work alongside clinicians.

*In our service*,* a lot of our budget went to hiring interpreters… it’s important to recognize the interpreter is a person… there’s a transference with that person. (LS)*

Their role was described as central—not only for communication but for building trust, navigating transference, and offering culturally grounded support. Overall, participants emphasized that bureaucratic systems—through rigid policies, limited funding, and institutional oversight—constrain efforts toward culturally responsive care, underscoring the need for systemic reform alongside individual training improvements.

### Shifting approaches to MH treatment

Several participants noted that shifting how MH treatment is approached could help clinicians better serve diverse populations. YJ stated, “sometimes, there’s a confusion between MH and mental illness” and this confusion must call clinicians to become better equipped to debunk such myths. GM advocated for adopting a positive psychology framework to help clients, which might reduce the stigma surrounding MH care:

*More a positive psychology framework of helping them feel that they can regain some form of control*,* a sense of competency.*

This perspective was supported by others who also stressed the importance of clinicians cultivating a participatory-based approach to treatment:

*It’s our role to create this atmosphere to encourage them to talk… This is a participatory positioning*,* not an ideological position. (SM)*

KT acknowledged the progress made in the MH sector regarding diversity and inclusion initiatives, particularly focusing on equity, diversity, and inclusion (EDI) efforts:

*I think the greater focus there has been in recent years on EDI and creating specifically safe spaces for these groups even within the broader range of services that are offered.*

Peer involvement was also identified as a valuable mechanism for reducing stigma and creating more inclusive services. Some organizations have adopted peer-informed approaches, involving clients in co-design and co-delivery to amplify lived experience and support culturally safe care.

*In our organization*,* we are peer positive. We do co-planning*,* co-design*,* and co-deliver*,* so the client’s voice is already there. (JX)*

These EDI initiatives were noted to enhance inclusivity both for clinicians—through improved training and organizational awareness—and for clients, by creating safer, more affirming service environments. Such a shift could help reduce the stigma faced by diverse communities in seeking MH care and foster a more supportive environment.

### Self-care and ongoing cultural learning

Participants emphasized the need for clinician self-care and ongoing cultural learning. SM noted, “It’s very important to think about self-care if you work in the system.”.

*Make sure you have a lot of your own support. You’re not there to fix them. Learn more about their communities. (NC)*

RJ highlighted the importance of self-reflection and humility, suggesting that clinicians who prioritize their own healing and curiosity are better attuned to clients’ needs:

*Just work on your own healing. Work on your humility*,* on your curiosity… if you’re curious and aware and you work on your own healing*,* you will be able to attune to all the things that other people are bringing in. (RJ)*

YJ and KT acknowledged that while additional training can help clinicians understand their boundaries, developing a broader understanding of cultural competence is essential:

*[Clinicians] can request additional training about cultural awareness*,* cultural sensitivity*,* cultural competence*,* but it doesn’t mean you will be skilled enough to provide [treatment]*,* but you will be skilled enough to understand your boundaries. (YJ)*

*I think that culture of ongoing and continuous learning and improvement is important. It doesn’t end in graduate training*,* but I think having a robust foundation in cultural competence is important. (KT)*

Findings suggest that despite recent progress, continuous improvement and sustained focus on cultural competence is necessary for the MH sector to adequately serve diverse populations.

## Discussion

This study reports on the subjective lived experiences of 19 clinical professionals treating socio-culturally diverse clients and recommendations for improvement. While many barriers discussed by clinicians are well documented in research and policy, this study reaffirms that they continue to persist in clinical practice, particularly when serving diverse populations. Despite increasing awareness, clinicians continue to face systemic and cultural challenges that hinder access to equitable care. This disconnect between growing awareness and ongoing service inequities reveals a critical gap between policy ideals and the lived realities of clinicians and clients alike. These enduring barriers highlight the need for deeper understanding and actionable solutions that bridge the gap between policy ideals and on-the-ground realities.

### Barriers to accessing and providing care

Clinicians identified key logistical barriers to MH services including financial constraints, long wait times, transportation challenges, and language access issues—consistent with prior studies [12, 16]. Financial barriers affect both clients, who often cannot afford treatment [19] and providers, with underfunded services leading to staffing shortages and low salaries [17]. These dual pressures suggest the need for structural reform, including expanded insurance, community-based funding, and sustainable financial models that support both access and retention. Long wait times and insufficient staffing delay care, especially for acute cases, sometimes prompting ineffective coping. Transportation challenges persist, particularly in underserved areas, with virtual care and transit supports offering partial solutions [3, 19]. Language barriers notably affect newcomers; although interpreter services exist, lack of mental health training for interpreters and discomfort with third-party disclosure reduce effectiveness [16, 18]. Bilingual staff and cultural mediators can help, but gaps remain, especially for racialized and senior populations. These logistical obstacles are often compounded by broader systemic inequities and cultural barriers, producing layers of exclusion that disproportionately impact already marginalized communities.

### Cultural and social barriers to MH service utilization

Stigma remains deeply embedded in many cultures. Clinicians noted that clients from racialized and immigrant communities often view therapy as shameful or inappropriate, a perspective rooted in cultural values that frame MH issues as weakness or moral failure [13]. In some cultures, MH challenges are seen as private family matters, and seeking external support can be perceived as a failure of personal or familial resilience [6, 15]. Clinicians (JP, SM, AN, TH, NS) observed that shame-based traditions in some cultures heighten fears of judgment, deterring care-seeking. NS remarked, “the population I work with. they’re coming from a shame-based culture.” Supporting this, several researchers have noted that seeking professional help is often framed as weakness, reinforcing shame, internalized stigma and delaying necessary interventions [7, 13]. While MH related stigma is not limited to non-western culture, culture-based stigma was a common barrier reported by the clinicians in this study.

Mistrust in MH systems also deterred clients, consistent with findings on systemic racism and lack of cultural humility in healthcare [12, 16]. Clinicians noted that personal and vicarious experiences of racism and stigmatization contributed to skepticism about Western MH practices. Clients fear being misunderstood or dismissed by providers unfamiliar with their culture, while concerns about confidentiality often deter them from seeking care. These findings align with Salam and colleagues who highlight how confidentiality concerns within medical and legal systems can discourage engagement with MH services [3]. Results further suggest that younger clients in particular need clear explanations about confidentiality policies to alleviate fears of exposure within their communities. The intersection of confidentiality concerns and mistrust highlights the importance of culturally responsive communication, where transparency and humility become essential tools for building trust. These findings reinforce calls for participatory approaches and community co-design in MH service delivery [20].

Family and community expectations—less examined in the literature—emerged as significant barriers. Clinicians noted that generational divides shaped MH attitudes: younger clients were more open to therapy, while older generations often saw MH concerns as spiritual or private. Concerns about family reputation and burdening others discouraged help-seeking, particularly in certain cultures where individual needs are subordinated to group well-being [3]. Some clinicians reported that integrating culturally responsive practices—such as involving family members, religious leaders, or traditional healers—can improve engagement [18]. Still, they emphasized the difficulty of balancing cultural respect with evidence-based care.

### Clinician recommendations

There is a notable gap in culturally responsive training, with clinicians often feeling unprepared to serve diverse populations due to Western-centric models that overlook culturally specific MH views [11, 16]. Participants highlighted the need for more practice-based training. This aligns with critiques of traditional evidence-based models, which may neglect the complexity of real-world clinical contexts [19, 20]. Research supports integrating hands-on experience with theoretical instruction to build clinician competency [4]. This includes not only training clinicians, but also preparing interpreters, cultural brokers, and other support personnel who serve as critical bridges between systems and communities. Participants called for shifts in MH treatment frameworks to challenge stigma and cultural misconceptions by centering lived experience and client-informed, participatory approaches [10, 13]. While equity, diversity, and inclusion efforts exist, inconsistent implementation highlights the need for sustained structural reforms [3]. Clinician well-being also emerged as a critical concern, particularly in settings where systemic pressures and cultural tensions intersect. Burnout and secondary trauma are common among MH professionals working with marginalized groups [14]. Cultural humility requires continuous self-reflection and deep understanding of intersecting social contexts, beyond checklist-style training [4, 11, 16]. Piazza and colleagues similarly argue that cultural competence is a dynamic, ongoing process rather than a fixed skill set [5]. Participants also challenged the sufficiency of additional training alone, critiquing static, checklist-style models of cultural competence [4]. Instead, they emphasized that meaningful cultural responsiveness emerges from sustained relational engagement, structural support, and a commitment to learning from clients and communities.

### Strengths and weaknesses of the study

A key strength of this study lies in its detailed, practice-based perspectives, gathered from frontline clinicians actively working with diverse populations. Their insights provide a nuanced understanding of how systemic and cultural dynamics intersect in everyday clinical work. The study also contributes to the literature by highlighting underexplored barriers, such as generational divides, family reputation, and clinician burnout in multicultural contexts. However, the study has several limitations. It reflects the experiences of a specific group of MH practitioners and may not be generalizable across all clinical or geographic settings. The exclusive focus on clinicians means that client perspectives—which are essential to understanding barriers from a service user standpoint—are absent. Additionally, while the study identifies areas for structural and educational improvement, it does not measure the effectiveness of culturally responsive practices in improving outcomes. The lack of gender diversity among participants (all identified as women) may also limit the scope of perspectives on MH practice.

#### Relation to other studies and key differences

Many of the barriers identified in this study mirror findings from prior research, including financial inaccessibility [8], stigma in racialized and immigrant communities [6, 13], and systemic mistrust due to racism or cultural misunderstanding [12, 16]. Like Salam and colleagues [3], this study found that logistical barriers such as transportation and language access remain persistent and often intersect with cultural concerns. Where this study extends existing literature is in its examination of family and community dynamics as active barriers to care. While other studies acknowledge stigma, clinicians here emphasized how multigenerational conflict and concerns about family reputation can impede care-seeking—a nuance less frequently explored. Additionally, participants discussed the emotional and ethical strain of navigating between cultural respect and evidence-based treatment—an area often underrepresented in policy discourse. The findings also add to critiques of conventional MH education [11, 20], by pointing to administrative and structural burdens that diminish clinician capacity and well-being. While previous research calls for cultural competence training, this study critiques one-off, checklist-style models and advocates for continuous, practice-based learning. It also echoes emerging scholarship [2, 7], calling for deeper integration of cultural humility, systemic awareness, and clinician self-care into MH systems.

#### Implications and conclusions

MH systems continue to fall short in adequately serving socioculturally diverse populations, particularly those facing systemic and structural barriers to care. Drawing on clinicians’ insights, this study identifies pressing challenges and points to concrete strategies for improving equity in service delivery. Findings have practical relevance, highlighting concrete steps such as embedding culturally responsive practices in clinical training, policies, and organizational protocols. For example, professional training programs would benefit from relevant culture focused curriculum along with hands on practical training working with clients from diverse socio-cultural backgrounds [27]. For policymakers, the findings highlight the urgent need for system-level reforms that address enduring obstacles such as financial constraints, stigma, mistrust, and prolonged wait times [6, 13, 16]. A central policy implication is the imperative to embed cultural responsiveness into all levels of MH infrastructure—education, clinical practice, and organizational policy. Clinicians must be supported through training and resources that enhance their capacity to build trust, navigate cultural complexity, and counter stigma using approaches rooted in humility and reflective practice [7, 11]. Supporting clinicians through targeted hands-on training, reflective practice, and self-care resources can enhance their capacity to deliver culturally responsive care and reduce burnout. For both clinicians and policymakers, it is critical that MH programs actively center client perspectives and address structural barriers such as language access and transportation. Integrating client perspectives into program design ensures interventions are culturally grounded, actionable, and responsive to community needs. Tokenistic inclusion must give way to meaningful organizational transformation that reflects the lived realities of diverse communities [3]. Thus, MH programs must actively integrate client perspectives to ensure interventions are culturally grounded and address structural barriers like language access and transportation. Addressing these barriers provides clear, implementable pathways to more equitable care. Institutional investments should also focus on reducing administrative overload and improving service integration. While clinician advocacy is valuable, sustainable, system-level supports are essential for long-term change [15, 16, 20].

Future research should examine how organizational structures—such as administrative bureaucracy and funding models—affect clinicians’ ability to provide culturally responsive care. Longitudinal mixed-method designs could evaluate how family-inclusive and community-based interventions reduce stigma over time. Research should also investigate the impact of clinician-client cultural matching and sustained professional development in cultural humility on both clinician burnout and client outcomes across diverse service contexts. Additionally, client-informed research is needed to complement clinician perspectives, revealing barriers and facilitators that may be overlooked in professional accounts. By emphasizing cultural responsiveness, clinician self-care, ongoing self and systemic reflection, this research lays the groundwork for future efforts to improve access to MH services for marginalized groups, contributing to the broader goal of achieving MH equity.

## Supplementary Information

Below is the link to the electronic supplementary material.

Supplementary Material 1

## Data Availability

The data is not publicly available due to the sensitive nature of qualitative data. Contact the corresponding author to discuss data availability.
