# Supplementary material for: Barriers to access, pathways to equity: clinicians’ perspectives on mental health service delivery
Source: BMC Health Serv Res. 2026 Jan 8;26:181. doi: 10.1186/s12913-025-13948-3 (PMC12874656; doi:10.1186/s12913-025-13948-3)
Supplement: Supplementary file 1 — Supplementary Material 1 [file 12913_2025_13948_MOESM1_ESM.docx]

**Supplementary Material: Interview Questions**

*Do you have any questions before we begin?*

**Clinician related questions**

1. Can you tell me a little bit about yourself?
2. Are there any aspects of your identity that you think impact your role as a clinician and the clients that you work with?
3. Is there anything you would recommend being included in graduate training that would've better prepared you for current role?
4. What is your current role within the organization?
   - Are you involved in any treatment design or program evaluation?
5. What motivated you to apply for a position in your current organization?
6. What do you perceive is your organization’s reputation as a mental health organization supporting equity-deserving groups in the broader community?

**Questions specific to your agency/program.**

1. Please provide us with a brief summary of the organization/agency culture (to the best of your ability) and how it led to developing/adapting the tailored program for equity-deserving individuals.
2. Please specify how or in what ways your programs meet the unique needs of the demographic you cater to.
3. What is your organization’s linguistic capacity to work with clients for whom English is not their first language?
4. How does your agency/programs commit to protecting client confidentiality and/or assure limiting disclosure of information?
5. What are some logistical and economic barriers that you have encountered while supporting clients from diverse sociocultural backgrounds?
6. How do you envision the organization evolving over time to meet the needs of the equity-deserving group?

**Questions related to Current Needs in Mental Health Services**

1. What is your understanding of the mental health and social service needs of different equity-deserving groups in your community?
2. What do you think are the opportunities to improve service over time?
   - What do you think you would need *in your profession* in order to more successfully support diverse groups/families (if applicable)?
3. What are some primary barriers (logistical, economic, service-related) that you have identified in supporting individuals of diverse sociocultural backgrounds?
4. Can you think of any ways that services have changed over time to successfully support families/individuals who are part of equity-deserving groups with diverse needs?
5. What advice would you give to someone who is starting a clinical job that requires providing mental health support to diverse clients?
6. How would you recommend continuing engagement with clinicians who support diverse community members and their families in the future?
7. Is there anything else that you would like to share with us about your experience working with diverse clients?
